# High school science fair and research integrity

**DOI:** 10.1371/journal.pone.0174252

**Published:** 2017-03-22

**Authors:** Frederick Grinnell, Simon Dalley, Karen Shepherd, Joan Reisch

**Affiliations:** 1 Department of Cell Biology, UT Southwestern Medical Center, Dallas, Texas, United States of America; 2 Department of Physics, Southern Methodist University, Dallas, Texas, United States of America; 3 Plano Independent School District, Plano, Texas, United States of America; 4 Department of Clinical Sciences, UT Southwestern Medical Center, Dallas, Texas, United States of America; VU University Amsterdam, NETHERLANDS

## Abstract

Research misconduct has become an important matter of concern in the scientific community. The extent to which such behavior occurs early in science education has received little attention. In the current study, using the web-based data collection program REDCap, we obtained responses to an anonymous and voluntary survey about science fair from 65 high school students who recently competed in the Dallas Regional Science and Engineering Fair and from 237 STEM-track, post-high school students (undergraduates, 1st year medical students, and 1st year biomedical graduate students) doing research at UT Southwestern Medical Center. Of the post-high school students, 24% had competed in science fair during their high school education. Science fair experience was similar overall for the local cohort of Dallas regional students and the more diverse state/national cohort of post-high school students. Only one student out of 122 reported research misconduct, in his case making up the data. Unexpectedly, post-high school students who did not participate in science fair anticipated that carrying out science fair would be much more difficult than actually was the case, and 22% of the post-high school students anticipated that science fair participants would resort to research misconduct to overcome obstacles. No gender-based differences between students’ science fair experiences or expectations were evident.

## Introduction

Research misconduct including fabrication, falsification, and plagiarism has become an important matter of concern in the scientific community [1]. Approximately 2% of researchers admit to committing misconduct at least once and 14% indicate they know of such behavior by colleagues [2]. Moreover, a marked increase in retraction rates of published papers can be attributed in part to instances of research misconduct [3]. Precise reasons why scientists commit misconduct vary greatly but include factors such as the conflicting pressures researchers experience and their perceptions about the fairness of the research enterprise [4]. Educational efforts aimed to increase research integrity have focused primarily on graduate students and postdoctoral fellows [5,6]. However, if research misconduct begins earlier in the science education curriculum, then introduction of education about responsible conduct of science would be valuable to incorporate earlier as well [7].

By bringing together the elements of problem selection, experimental design, implementation, analysis and communication of research findings, science fair presents one of the few opportunities in science education for students to experience themselves the overall practices of science, an opportunity often not available in the science classroom setting. Consequently, science fair can serve as a primary means of introducing and teaching students about how to conduct authentic scientific research [8–10]. According to the *Science Buddies* website [11], 9 million students from around the world currently participate annually in some version of science fair, which includes the Science Talent Search and other major science fair competitions (e.g., Google, Siemens).

In anonymous surveys, high school students openly admit to cheating [12,13]. How about cheating in science fair? Research misconduct was the subject of a 2011 Intel International Science and Engineering Fair finalist project entitled, *Science Un-Fair*?: *A Reformative Analysis of Parental Factors and Research Misconduct*. The project consisted in part of a survey by student researchers who reported that “at least 60% of the entire population of MST researchers [students at duPont Manual Magnet High School] committed acts of scientific misconduct” [14]. Exactly what this meant was unclear. According to a news summary about the project, 20% of the students “had altered their hypothesis after finishing their study, and 33% had abused the scientific method in some other way”[15]. Altering one’s hypothesis in response to the data is not good science fair practice because, as a model of the scientific method, students participating in science fair are asked to use their data to confirm or refute a starting hypothesis. In actual practice of science, however, altering one’s hypothesis in response to the data is not unusual. Indeed, hypotheses are difficult to refute because of the problem distinguishing between whether the hypothesis is wrong or the method used to test the hypothesis is inadequate [16].

The only published, peer-reviewed study about cheating in high school science fair involved 24 students (7th-11th grade) who were recruited from the year 2000 Bell Montreal Regional Science Fair. These students had been required to carry out a high school science fair project. Five admitted in anonymous surveys to making up their research data [17]. The aim of the current study was to conduct a more robust analysis of the extent of research misconduct in high school science fair based not only on the experiences of high school students, but also on post-high school students engaged in STEM-related career paths.

## Materials and methods

Study design entailed administering a voluntary and anonymous online survey to students. This study was approved by the UT Southwestern Medical Center (UTSW) IRB. The survey was carried out with the REDCap survey and data management tool managed by the biostatistics group in the UTSW Department of Clinical Sciences.

We surveyed four different student groups. One test group consisted of high school students (grades 9–12) who recently had participated in the Dallas Regional Science and Engineering Fair (DRSEF). These students were mostly from a suburban district with a strong commitment to science education that encourages but does not require students to carry out a science fair project. For the high school student group, informed consent was obtained by contacting students’ parents (699) after the science fair was over. Students whose parents gave consent (112) were sent an email with a public link to the survey and an access code meant to prevent duplicate submissions.

The other three test groups were students doing research in one of the programs at UTSW: undergraduate college students doing summer research as part of the Summer Undergraduate Research Fellows program; 1st year graduate students in the biomedical sciences; and 1st year medical students doing a summer research project. The students at UTSW come from all over Texas and the United States. Informed consent was judged not to be required for the three older student groups. Email addresses of these students were provided to REDCap management by the program offices, and REDCap sent the surveys directly to all of the students participating in the programs.

Survey content was adapted from the Montreal study [17]. The survey included questions about student demographics (gender, educational level and program); type of science fair participation (individual/team; required/not required); help expected and received; and obstacles encountered and solutions implemented to overcome obstacles. DRSEF and UTSW graduate/medical student surveys are shown in supporting information (S1 and S2 Surveys, respectively). The undergraduate student survey began with different demographic questions compared to the graduate/medical student survey but otherwise contained the same content. The draft survey document and procedures were piloted successfully with graduate students in the fall of 2014 and used subsequently. Student responses from the pilot study were included in the analyses.

Most respondents completed all of the survey questions, and the dataset for this study can be found in supporting information (S1 Dataset). Frequency counts and percentages for the data items were tabulated and sorted. Data shown in the figures are organized by descending frequency counts based on the high school respondent group and presented both graphically to make overall trends easier to appreciate and tabularly to present actual numbers. Significance of answers comparing different groups was analyzed using Chi-square contingency tables. Where p values 0.05 or lower were determined, significance is indicated by the editorial symbol § in the graphs and by asterisks in the tables including the p values.

## Results

### Demographics

Tables 1 and 2 provide information about the students who participated in the survey. Response rate ranged from 58% for high school students to 25% for graduate students. Although some variability in gender balance of the respondents occurred, e.g., more girls than boys for undergraduate students and more boys than girls for medical students, the differences were not significant statistically. Of the post high school students, 20–25% (57 total) carried out a high school science fair project. For most questions about science fair experience, we compared the 65 high school students (32 girls, 32 boys, 1 no gender selection) with the 57 post high school students who had carried out high school science fair (23 girls, 29 boys, 5 no gender selection). These groups are designated HS and PHSy respectively. The post high school students who had not participated in high school science fair, designated PHSn, consisted of 180 students (92 girls, 78 boys).

**Table 1:** Survey demographics.

Student Group	High school students	Undergrad students	Medical students	Graduate students
Survey Feature				
# Students Sent Surveys	112	324	196	107
# Students Responded (%)	65 (58.0)	148 (45.6)	62 (31.6)	27 (25.2)
# Girls^1^ (%)	32 (49.2)	82 (55.4)	28 (45.1)	5 (41.7)^1^
# Boys^1^ (%)	32 (49.2)	66 (44.6)	33 (53.2)	7 (58.3)^1^
# Participated in High School Science Fair (%)	65 (100)	38 (25.7)	13 (21.0)	6 (22.2)

^1^Not all students indicated gender, and gender choice not an option in the graduate student pilot survey.

**Table 2:** Type of science fair project and requirement.

Student Group (#)	High School Girls (32)	High School Boys (32)	All High School Students (65)	Post High School Girls (23)	Post High School Boys (29)	All Post High School Students (57)
Survey Feature						
% Individual Project	65.6	78.1	72.3	69.6	79.3	73.7
% Team Project	34.4	21.9	27.7	30.4	20.7	26.3
% Science Fair Required	9.4	6.3	7.7	34.8	44.8	40.4
% Science Fair Optional	43.8	53.1	49.2	39.1	41.4	42.1
% Science Fair Optional but Satisfied School Project Requirement	46.9	40.6	43.1	26.1	13.8	17.5

Most students who participated in science fair (~72% overall) carried out individual projects. When asked about science fair requirements, high school students (>90%) reported that they were not required to do science fair but almost half (43% overall) used science fair to fulfill a school project requirement. On the other hand, ~40% overall of the post high school students were required to do science fair, and less than 20% overall used science fair to fulfill a school project requirement. Differences between boys and girls in the high school and post high school groups were apparent but not significant statistically.

### Help

**Fig 1:**
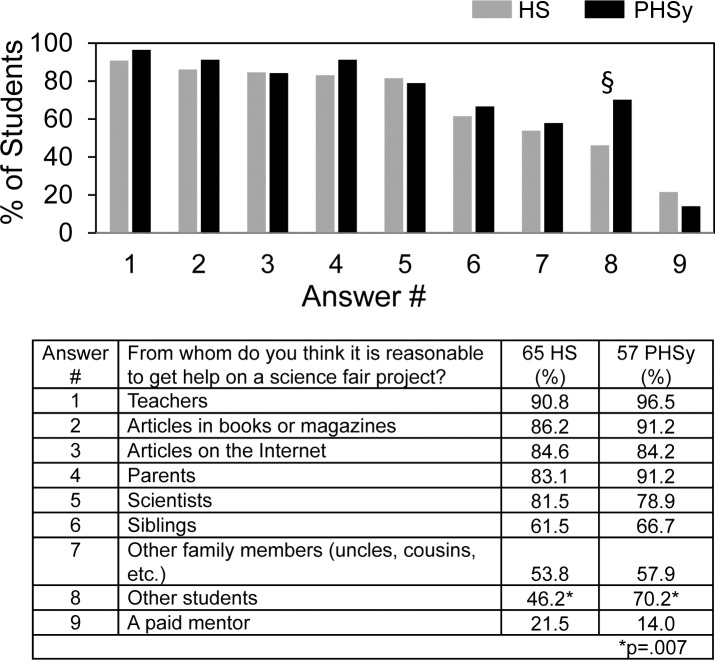
From whom do you think it is reasonable to get help on a science fair project?

Fig 1 shows student attitudes regarding from whom it would be reasonable to receive help. Teachers, articles, and parents were selected with the highest frequency. Concerns have been raised about the fairness of science fair when students have access to scientist mentors and professional laboratory facilities [18,19]. Nevertheless, ~80% of the students selected scientists as a reasonable potential source of help in contrast to ~20% or less who selected paid mentors. Comparing HS and PHSy students, the only significant difference was that more PHSy students thought it reasonable to receive help from other students.

**Fig 2:**
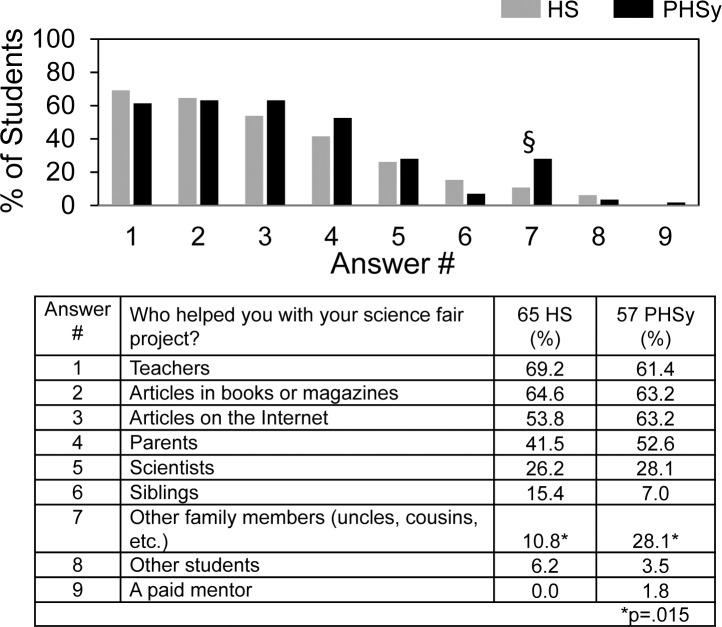
Who helped you with your science fair project?

Fig 2 shows student answers to the practical question about from whom they actually obtained help. For both HS and PHSy students, teachers, articles, and parents were to a similar extent the main sources of help they received. Only 26–28% of the students reported receiving help from scientists. Compared to HS students, more PHSy students received help from other students.

**Fig 3:**
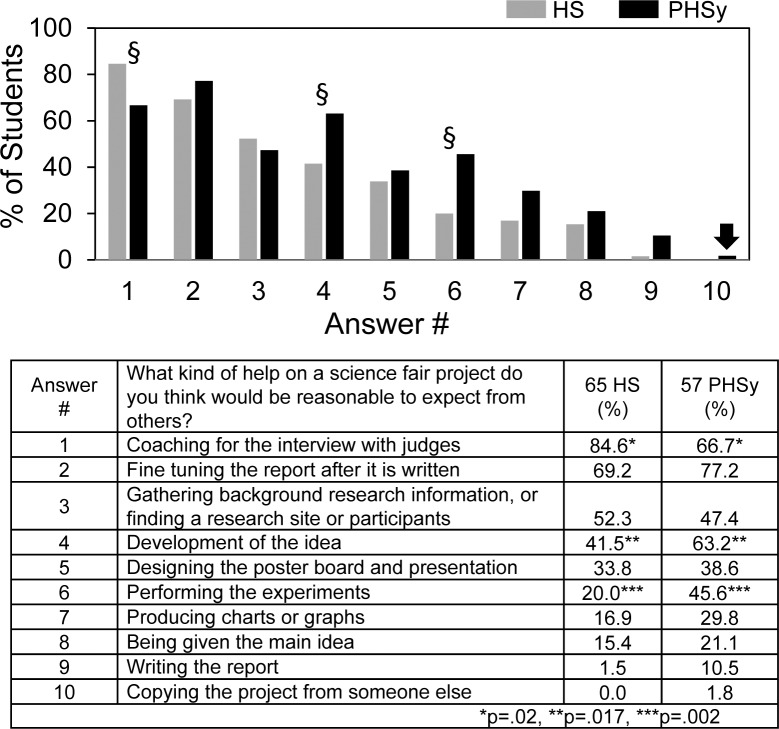
What kind of help on a science fair project do you think would be reasonable to expect from others?

Fig 3 shows student attitudes regarding what kind of help it would be reasonable to expect. Coaching for the interview, fine tuning reports, and gathering background information were selected most frequently. Although the *Science Buddies* website [11] advertises >1000 science fair project ideas in all areas of science, only ~20% or less of the students thought it reasonable to be given the main idea for their projects. One student (arrow) thought it would be reasonable to copy the project from someone else, which would amount to plagiarism. HS and PHSy showed significant differences in their expectations about coaching, development of the idea, and performing the experiments.

**Fig 4:**
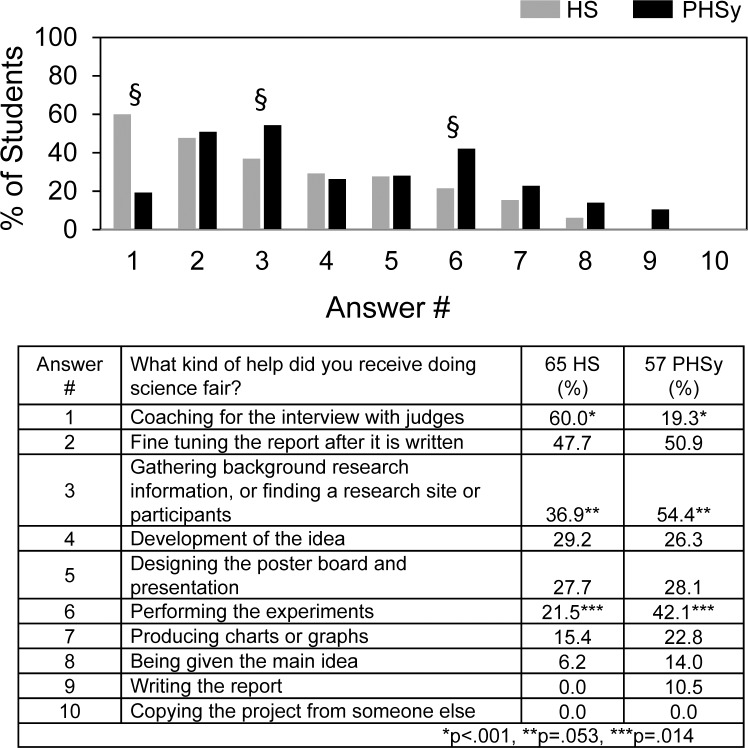
What kind of help did you receive doing science fair?

Fig 4 shows the types of help that students actually received. 60% of the HS students reported receiving coaching, which is a feature known to be emphasized for students competing in DRSEF. Otherwise, no more than 50% of the students received any particular type of help. PHSy students were more likely to receive help gathering background information and performing the experiments. Less than 10% of the students reported being given the main idea and none said that they copied their project from someone else, i.e., plagiarism. Four HS students and 2 PHSy students reported receiving no help at all, 26%-28% received one type of help, and 42%-46% received 2–3 types of help.

Notwithstanding what appeared to be a modest level of help both in terms of sources and types reported by the students (Fig 2 and Fig 4), Table 3 shows that when asked about their satisfaction with the kind and amount of help they received from teachers, ~2/3 or more of the HS and PHSy students reported that they were satisfied. One noticeable difference, PHSy boys reported lower levels for both kind of help (p = .005) and amount of help (ns, p>.05) compared to HS boys.

**Table 3:** Satisfaction with kind and amount of help received in science fair (all students and gender).

Student Group (#)	High School Girls (32)	High School Boys (32)	All High School Students (65)	Post High School Girls (23)	Post High School Boys (29)	All Post High School Students (57)
Survey Feature						
% Received the Kind of Help Needed from Teachers	75.0	87.6*	80.0	78.3	55.2*	64.9
% Received the Amount of Help Needed from Teachers	71.9	78.1	73.8	73.9	62.1	66.7

*p = .005.

### Obstacles

**Fig 5:**
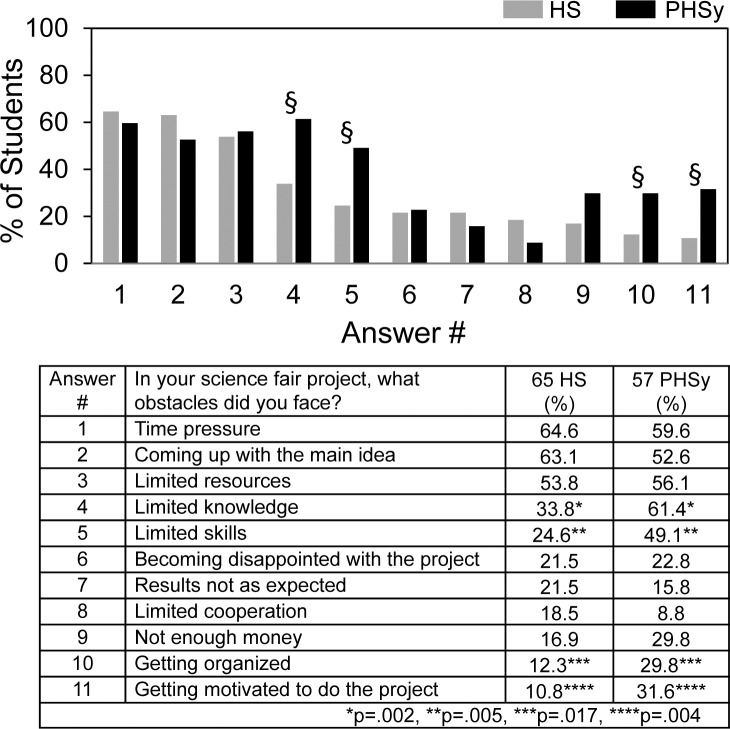
In your science fair project, what obstacles did you face?

Fig 5 shows student answers to questions about obstacles encountered in doing science fair. For HS students, time, coming up with the main idea, and limited resources were selected most often as the obstacles encountered. PHSy students also encountered these obstacles frequently. In addition, they reported experiencing limited skills and limited knowledge more so than HS students. Interestingly, only 16–22% of the students experienced results not as expected as an obstacle although overcoming unexpected findings frequently is part of everyday practice of science [7].

**Fig 6:**
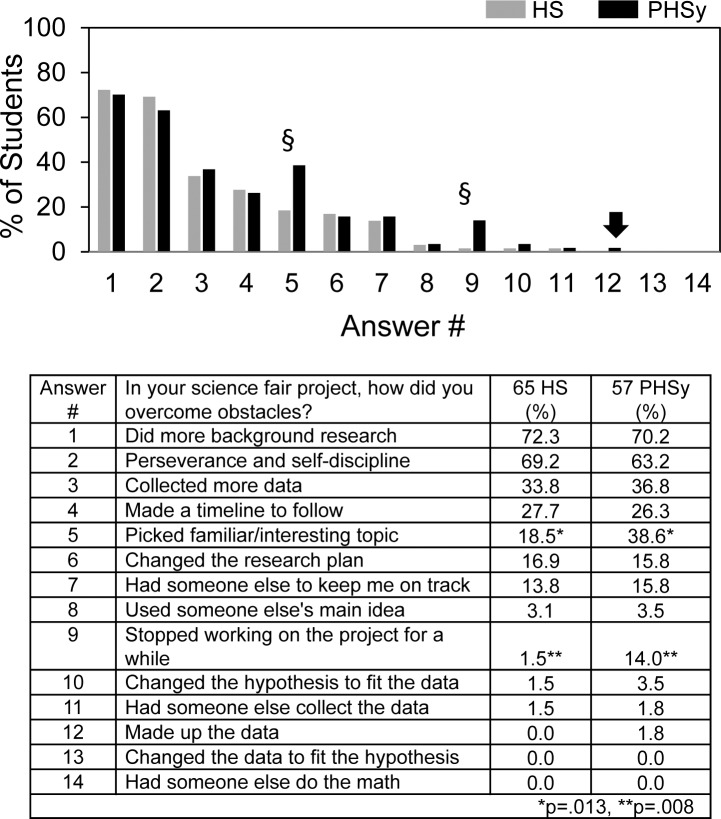
In your science fair project, how did you overcome obstacles?

Fig 6 shows student answers to questions about how they overcame the obstacles. Doing more background research and perseverance were selected most often as the means to overcome obstacles and used by more than 60% of the students. The other choices were selected by 40% or less. PHSy students reported more than HS students picking a familiar topic and stopping work on the project as means to overcome obstacles.

One PHSy student (arrow) reported making up his data as the means to overcome obstacles. This student was required to participate in science fair and carried out an individual project. He was part of the ~1/3 of students who reported not having received the kind or amount of help needed from teachers, although he did report receiving help from parents and siblings doing the experiments and fine tuning the report. Regarding number of obstacles encountered, he was at the extreme margin and checked all 11 potential obstacles. By contrast, the average HS and PHSy students reported encountering 3.5±1.9 (s.d.) and 4.2±2.3 (s.d.) obstacles respectively.

### Expectations about science fair by PHSn students

**Fig 7:**
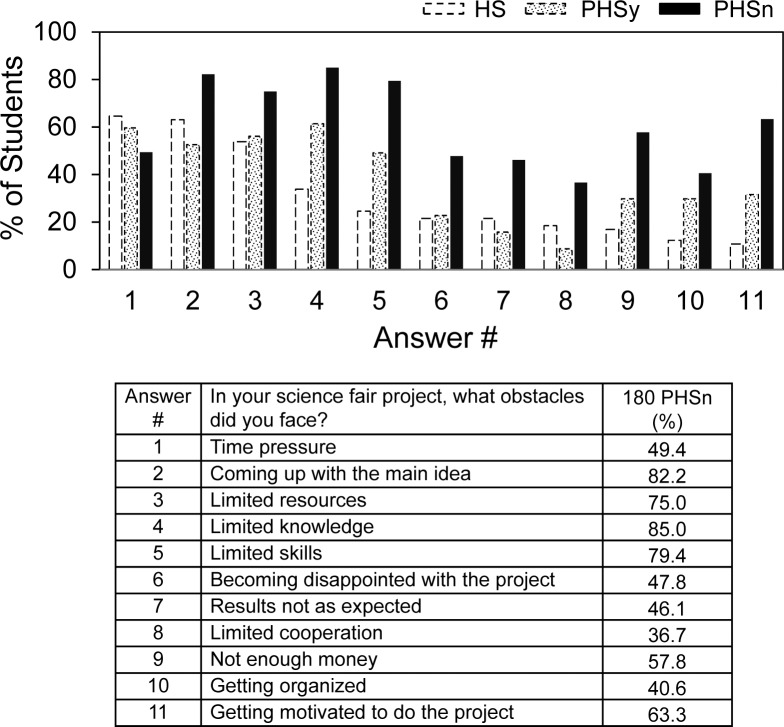
What obstacles do you think students who do science fair usually face?

PHSn students answered questions similarly as PHSy and HS students regarding from whom and what kind of science fair help (S1 and S2 Figs). However, when asked about obstacles, PHSn students imagined that many more obstacles would be encountered by science fair participants than actually was the case. Fig 7 shows that except for time pressure, PHSn student responses (solid bars) were higher for every obstacle encountered by HS and PHSy students (data from Figs 5 and 6 shown by dotted bars). Table 4 summarizes the extent of these differences and shows anticipated obstacles were much higher for PHSn compared to those actually encountered by PHSy and HS students.

**Table 4:** Obstacles that HS and PHSy students encountered vs. that PHSn students anticipated.

Student Group (#)	HS (65)	PHSy (57)	PHSn (180)
Survey Feature			
Average # Obstacles Encountered (HS, PHSy) or Anticipated (PHSn)	3.45	4.19	6.64
% of Total Obstacles Possible (11 obstacles = 100%)	31.3*	38.1**	60.3*^/^**

*p < .001

**p < .001.

**Fig 8:**
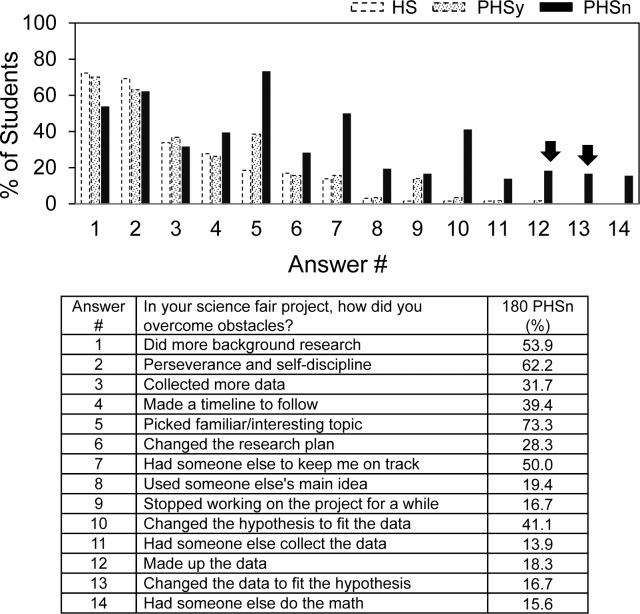
How do you think students who do science fair usually overcome obstacles?

Fig 8 shows the ways in which PHSn students anticipated that students who participated in science fair would overcome obstacles. Compared to HS and PHSy students, an increase in several categories was observed including picking a familiar topic, being kept on track by someone else, and changing the hypothesis to fit the data. Unexpectedly, however, behaviors corresponding to scientific misconduct also were anticipated by the PHSn students (arrows), i.e., making up the data and changing the data to fit the hypothesis. Overall, more than 20% of the PHSn students selected these categories, and Table 5 summarizes the findings. Of those who expected misconduct behavior, more than half anticipated both making up the data and changing the data to fit the hypothesis. About 40% of medical students in the PHSn group expected misconduct, which was twice as high as the percentage of undergraduate students in the PHSn group that expected misconduct.

**Table 5:** Misconduct anticipated by 40 out of 180 PHSn students.

Type of Misconduct Expected to be Carried Out.		# Students (%)	
Make up the data.		10 (25.0)	
Change the data to fit the hypothesis.		7 (17.5)	
Make up and change the data .		23 (57.5)	
Current Program of Those Who Anticipated Misconduct by Science Fair Participants	Undergrad Students	Medical Students	Graduate Students
# Students/Total (%)	21/110 (19.1)*	18/49 (36.7)*	1/11^1^ (9.1)

^1^Question not asked to students in the pilot study

*p = .017.

### Gender

The data analyzed in this study were sorted by gender. No differences between boys and girls in experiences or expectations were detected that reached statistical significance.

## Discussion

Research misconduct has become an important matter of concern in the scientific community [1], but the extent to which such behavior occur early in science education has received little attention [7]. Several studies have described scientific misconduct in relationship to undergraduate research, where getting the expected “right” answer in laboratory courses can become the driving factor for misbehavior [20–24]. Unlike the situation with undergraduates, only a single peer-reviewed study about misconduct in high school science fair has been published [17]. We initiated our research on science fair because gaining a better understanding of when problems with research integrity emerge is important not only for understanding the underlying causes, but also for deciding when it is appropriate to introduce education about research integrity into the science education curriculum.

The one peer reviewed study on research integrity in high school science fair involved 24 students (7th-11th grade) who were required to carry out a project, and who had competed in the year 2000 Bell Montreal Regional Fair [21]. We adapted our survey questions from the Montreal study and tested a larger and more diverse student sample consisting of high school students who had just participated in the Dallas Regional Science and Engineering Fair and post high school students–undergraduate, graduate, and medical students–doing research at UT Southwestern Medical Center. We included post high school as well as high school students for several reasons. First, most research on the high school science fair experience has focused on students still in high school, whereas the post high school cohort in our study had subsequently pursued STEM career interests. Second, most high school science fair studies have focused on single geographic groups. We were able to compare the experiences of students from a local geographic cohort (DRSEF participants) with the more diverse cohort of post high school students who come from all over Texas and the United States. Third, we anticipated that post high school students might be more open to sharing their high school science fair experiences compared to students who were still in high school and might have been reluctant to do so even though we told them the surveys were anonymous.

In the Montreal study, based on an anonymous survey, five of the students (21%) admitted to making up their research data [21]. Student responses in our surveys from 65 high school students and 57 post high school students showed that less than 1% of the students carried out research misconduct. Although we cannot exclude the possibility that students did not fully report misconduct, previous work on student cheating in high school and college has been mostly based on such anonymous and voluntary surveys. Besides being larger, our research population differed from the Montreal study in that the high school students we surveyed had mostly elected to do science fair (>90%) rather than being required to participate. Of the post high school students, ~40% had been required to participate in science fair, but these students were interested in science based on their subsequent STEM-related educational trajectories.

Fear of failure is a common explanation for cheating in high school [25] and was suggested by Syer and Shore to be a likely explanation for the science fair misconduct they observed in the Montreal study [17]. Competitive science fair typically does not value failed experiments and disappointing data even though these are common features of everyday practice of science [16]. In our study, the one undergraduate student who reported making up his data fit into a potential failure profile in that he encountered every potential obstacle in his project and did not receive the help that he expected from teachers. However, he did receive help from parents and siblings, whereas some students reported receiving no help from any source. Also, the one student in our survey who reported committing misconduct was required to do science fair as was the case for all the students in the Montreal study. Neither the Montreal survey nor ours asked students specifically about the fairness of a science fair requirement. This question will be important for future studies since a perception of lack of fairness has been implicated in research misconduct by scientists [4].

Overall HS students and PHSy students reported similar science fair experiences. Parents, teachers and articles (internet and books/magazines) were the primary sources of help for both groups and occurred to a similar extent. Fine tuning the report and gathering background information were important types of help received for both groups. In addition, receiving help coaching for the interview (HS students) and performing the experiments (PHSy students) stood out. Except for PHSy boys, ~75% or more of the students reported that they were satisfied with the kind and amount of help that they received from teachers. Limited time, coming up with the main idea, and limited resources were the major obstacles for both groups. PHSy students also reported limited knowledge and skills. Interestingly, only 20% of the students reported results not as expected as an obstacle, which is an important difference between science fair and practice of science since results not as expected as an obstacle is a common feature of the latter [20]. Finally, for both groups, more background research and perseverance were the main ways of overcoming obstacles. Given the broad similarities between the local high school group and the state/national post high school group, a reasonable conclusion is that student high school science fair experience in the US has developed a national character.

Unexpectedly, only 20–25% of the post high school students had carried out a science fair project. Students who had not participated in science fair anticipated that doing so would be much more difficult than was actually experienced by students who had participated. Whether these students don’t do science fair because their schools didn’t offer the opportunity or because they think it is too difficult or for some other reason should become clearer in our futures studies using a modified survey. Also unexpectedly, the PHSn students exhibited considerable skepticism about the honesty of students who competed in science fair with more than 20% anticipating scientific misconduct by making up the data or changing the data to match the hypothesis, and medical students were twice as likely as undergraduates to have this expectation. Whether the PHSn students actually knew of such behaviors by science fair participants or were just guessing also is a question for future studies. In any case, the finding that so many students anticipate research misconduct will be associated with science fair suggests that integrating some discussion of responsible conduct of science might be valuable at the high school level.

Finally, we observed no gender differences in student answers in our surveys regarding science fair experiences and expectations. This finding is consistent with other studies demonstrating the ongoing increased involvement of girls in science fair and equivalent success rates of girls and boys [26,27].

## Supporting information

S1 Fig Answers to the question, “From whom do you think it is reasonable to get help on a science fair project? ” (TIF)

S2 Fig Answers to the question, “What kind of help on a science fair project do you think would be reasonable to expect from others?” (TIF)

S1 Dataset Excel dataset containing all of the survey questions and answers described in this paper. (XLSX)

S1 Survey Survey questions used with DRSEF students. (PDF)

S2 Survey Survey questions used with UTSW graduate/medical students. (PDF)
