# Supplementary figures and images for: High school science fair and research integrity
Source: PLoS One. 2017 Mar 22;12(3):e0174252. doi: 10.1371/journal.pone.0174252 (PMC5362261; doi:10.1371/journal.pone.0174252)

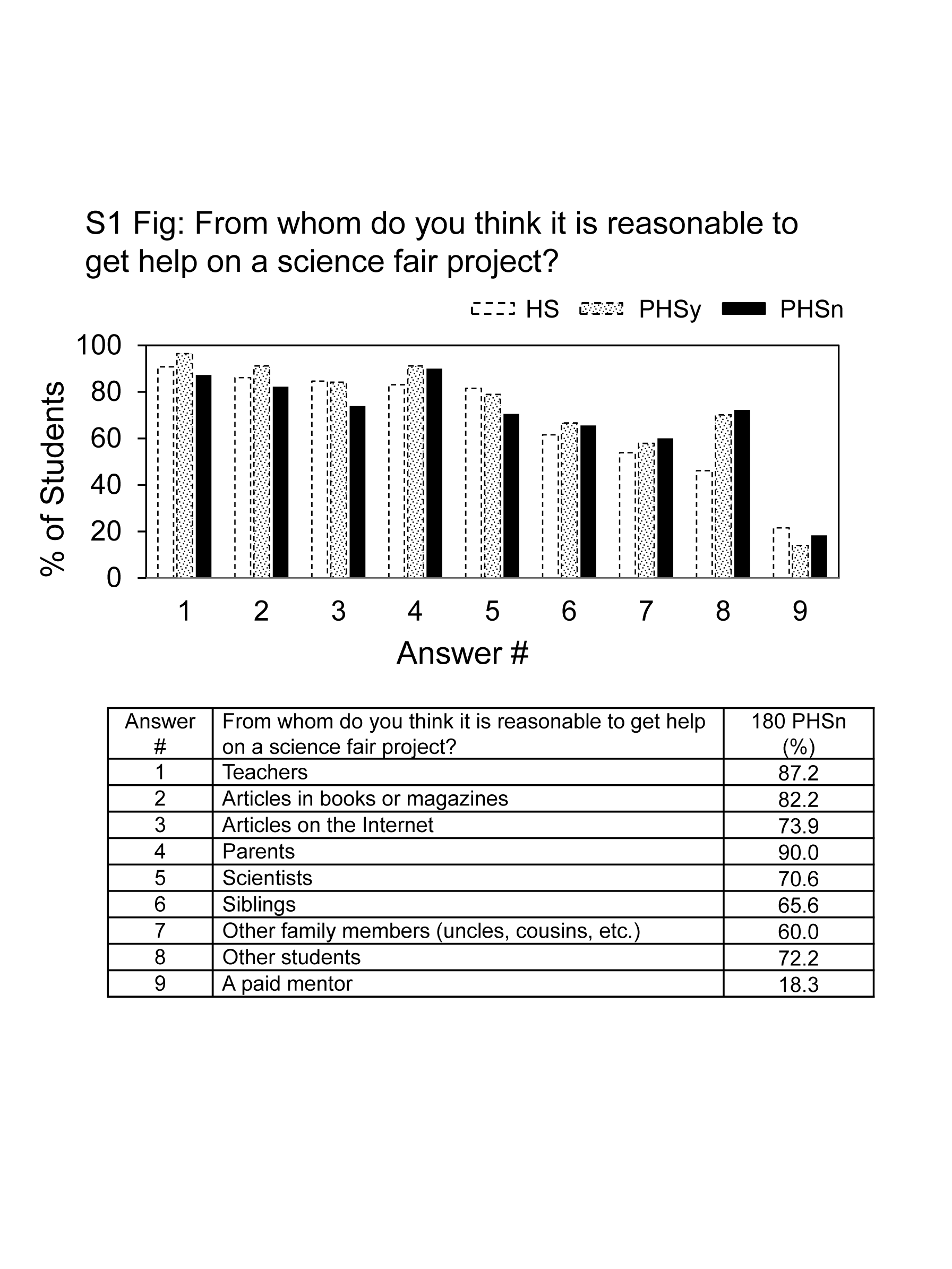

Supplement: S1 Fig — (TIF) [file pone.0174252.s001.tif]

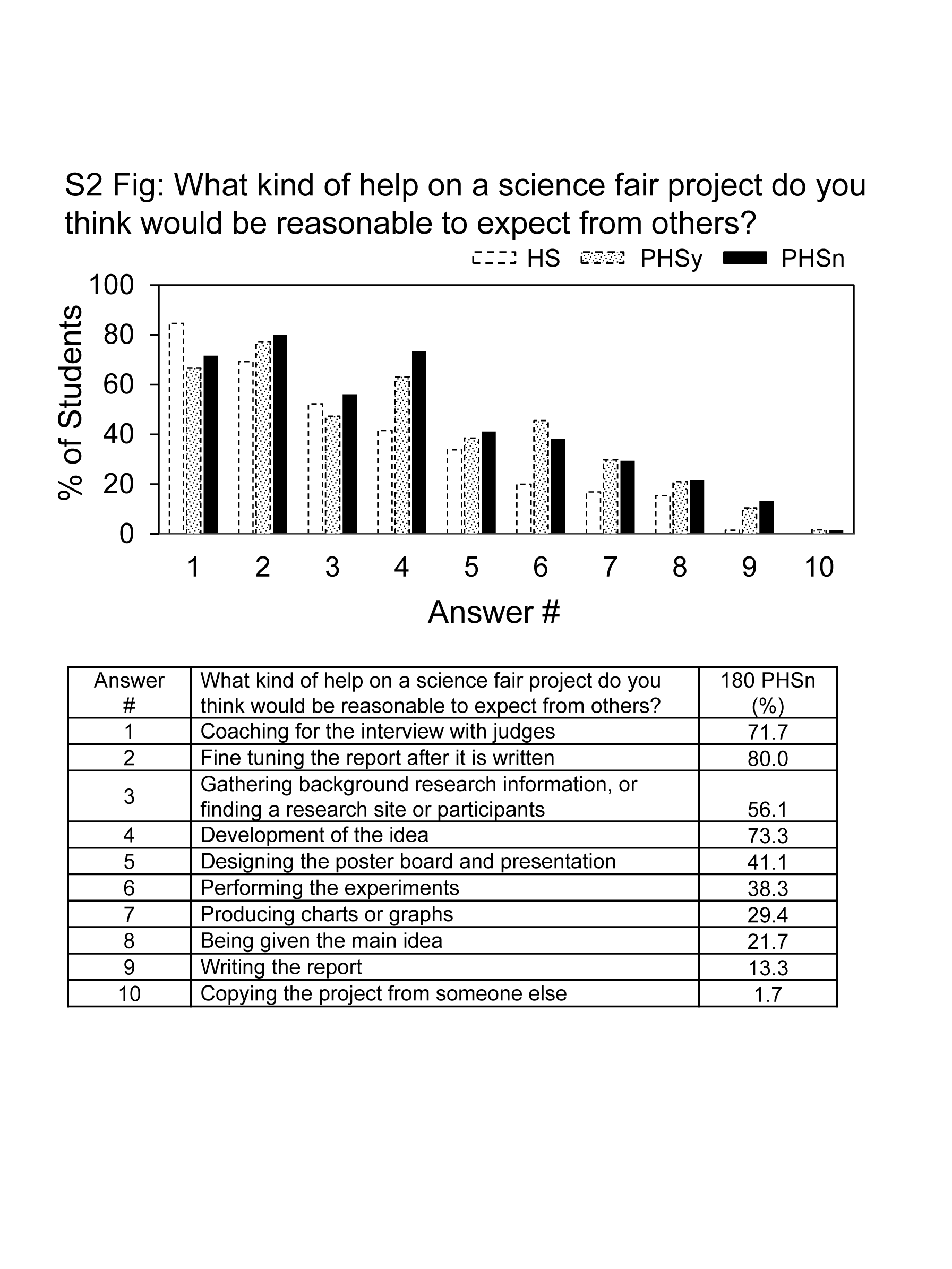

Supplement: S2 Fig — (TIF) [file pone.0174252.s002.tif]
