# Supplementary material for: High school science fair and research integrity
Source: PLoS One. 2017 Mar 22;12(3):e0174252. doi: 10.1371/journal.pone.0174252 (PMC5362261; doi:10.1371/journal.pone.0174252)
Supplement: S1 Survey — (PDF) [file pone.0174252.s004.pdf]

High School Science Fair Survey

We are interested in learning more about student experience with high school science fair. We would like to know about your recent experience and general impressions concerning what help it would be reasonable for you and other students to receive when doing science fair projects. Also, we would like to know what obstacles you faced in carrying out your project and how you overcame them. This survey, which consists of 17 questions, is completely anonymous. We are only interested in overall trends. You may leave blank any question you prefer not to answer. It is important that you give honest replies to those questions that you do answer since the results may be used to influence science fair practices in the future. Your participation in the survey is voluntary; however, the more students who participate in the survey, the more statistically persuasive will be any conclusions.

1. Enter your 5-digit survey access number ________________________________

2. What grade are you in?
   - ◯ 9th
   - ◯ 10th
   - ◯ 11th
   - ◯ 12th

3. Gender?
   - ◯ Female
   - ◯ Male

4. Was your science fair project Team or Individual?
   - ◯ Team
   - ◯ Individual

5. Were science fair projects required by your school?
   - ◯ Yes
   - ◯ No
   - ◯ No, but I did a science fair project to satisfy a school project requirement

6. Do you think science fair projects should be optional or required? (This need not be for science fair competition.)
   - ◯ Optional
   - ◯ Required

7. Reason why?

____

8. Do you think science fair projects for competition should be optional or required?
   - ◯ Optional
   - ◯ Required

9. Reason why?

____

10. From whom do you think it would be reasonable to receive help on a science fair project?
   - ☐ (1) Parents
   - ☐ (2) Siblings
   - ☐ (3) Other family members (uncles, cousins, etc.)
   - ☐ (4) Teachers
   - ☐ (5) Other students
   - ☐ (6) Articles on the Internet
   - ☐ (7) Articles in books or magazines
   - ☐ (8) Scientists
   - ☐ (9) A paid mentor
   - ☐ (10) Other

   (Check all that apply)

10a. Specify? ________________________________

11. In carrying out your project, who actually helped you?

- ☐ (1) Parents
- ☐ (2) Siblings
- ☐ (3) Other family members (uncles, cousins, etc.)
- ☐ (4) Teachers
- ☐ (5) Other students
- ☐ (6) Articles on the Internet
- ☐ (7) Articles in books or magazines
- ☐ (8) Scientists
- ☐ (9) A paid mentor
- ☐ (10) Other

(Check all that apply)

11a. Specify? ______________________________

12. What kind of help on a science fair project do you think would be reasonable to expect from others?

- ☐ (1) Being given the main idea
- ☐ (2) Development of the idea
- ☐ (3) Gathering background research information, or finding a research site or participants
- ☐ (4) Performing the experiments
- ☐ (5) Fine tuning the report after it is written
- ☐ (6) Writing the report
- ☐ (7) Designing the poster board and presentation
- ☐ (8) Producing charts or graphs
- ☐ (9) Coaching for the interview with judges
- ☐ (10) Copying the project from someone else
- ☐ (11) Other

(Check all that apply)

12a. Specify? ______________________________

13. In carrying out your project, what kind of help did you actually receive?

- ☐ (1) Being given the main idea
- ☐ (2) Development of the idea
- ☐ (3) Gathering background research information, or finding a research site or participants
- ☐ (4) Performing the experiments
- ☐ (5) Fine tuning the report after it is written
- ☐ (6) Writing the report
- ☐ (7) Designing the poster board and presentation
- ☐ (8) Producing charts or graphs
- ☐ (9) Coaching for the interview with judges
- ☐ (10) Copying the project from someone else
- ☐ (11) Other

(Check all that apply)

13a. Specify? ______________________________

14. In carrying out your project, did you get the kind of help you wanted from teachers?

- ○ Yes
- ○ No

14a. What kind of help would you have liked but did not receive?

____

15. In carrying out your project, did you get the amount of help you wanted from teachers?

- ○ Yes
- ○ No

16. In carrying out your project, what obstacles did you face?

☐ (1) Coming up with the main idea
☐ (2) Getting motivated to do the project
☐ (3) Becoming disappointed with the project
☐ (4) Limited resources
☐ (5) Limited knowledge
☐ (6) Limited skills
☐ (7) Limited cooperation
☐ (8) Getting organized
☐ (9) Time pressure
☐ (10) Not enough money
☐ (11) Results not as expected
☐ (12) Other
(Check all that apply)

16a. Specify? __________________________________

17. In carrying out your project, how did you overcome the obstacles you encountered?

☐ (1) Used someone else's main idea
☐ (2) Picked a familiar/interesting topic
☐ (3) Did more background research
☐ (4) Stopped working on the project for a while
☐ (5) Made a timeline to follow
☐ (6) Perseverance and self-discipline
☐ (7) Had someone else to keep me on track
☐ (8) Had someone else do the math
☐ (9) Changed the research plan
☐ (10) Collected more data
☐ (11) Had someone else collect data
☐ (12) Made up the data
☐ (13) Changed the hypothesis to fit the data
☐ (14) Changed the data to fit the hypothesis
☐ (15) Other
(Check all that apply)

17a. Specify? __________________________________
